# Root canal shaping of curved canals by Reciproc Blue system and Pro Taper Gold: A micro-computed tomographic study

**DOI:** 10.4317/jced.57180

**Published:** 2021-02-01

**Authors:** Rogério-Vieira Silva, Murilo-Priori Alcalde, Martinho-Campolina-Ribeiro Horta, Clarissa-Teles Rodrigues, Frank-Ferreira Silveira, Marco-Antônio Duarte, Eduardo Nunes

**Affiliations:** 1 Department of Dentistry, Pontifical Catholic University of Minas Gerais, Belo Horizonte, Minas Gerais, Brazil; b) Northeastern Independent College / FAINOR, Vitória da Conquista, Bahia, Brazil; 2 Department of Dentistry, Endodontics and Dental Materials, Bauru School of Dentistry, University of São Paulo, SP, Brazil; 3 Department of Dentistry, Pontifical Catholic University of Minas Gerais, Belo Horizonte, Minas Gerais, Brazil

## Abstract

**Background:**

This study aimed to evaluate the centralization and transportation of ProTaper Gold (PTG) rotary system and Reciproc Blue (RB) reciprocating system in curved canals, by using micro-CT.

**Material and Methods:**

Twenty extracted mandibular molars were previously scanned by using the SkyScan 1174 microtomograph to select the Vertucci IV anatomic type. The specimens were divided into two groups (n=10) according to the mechanized system used to prepare the root canals. The teeth were scanned by micro-CT to calculate the increase volume, percentage of dentin removed, remaining dentin thickness, structure model index (SMI), degree of transportation and centering ability of root canals. The Student’s t test was used to evaluate differences between PTG and RB in each measurement evaluated.

**Results:**

No significant differences were found between the groups in the increase of the total root canal and apical volume; percentage of dentin removed after preparation; SMI of the mesiolingual canal; degree of transportation of the canal and centering ability of the cervical and middle thirds (*P*>0.05). There were significant differences in the mesiobuccal canal in SMI and in the centering ability of the apical third (*P*<0.05). Concerning the remaining dentin thickness, there was also no significant diferences between the groups, except for some regions were RB was observed to have a superior cutting capacity (*P*<0.05).

**Conclusions:**

Both systems were efficient and safe for performing preparation of the moderately curved root canals of mandibular molars. RB instruments produced more circular and better centralized canals in the apical third of the mesiobuccal canal, with superior cutting action when compared with PTG instruments.

** Key words:**Nickel-titanium instrument, heat treatment, micro-computed tomography, canal transportation

## Introduction

Root canal shaping is a fundamental stage for achieving clinical success in endodontic therapy (1). Therefore, the presence of accentuated angle and curvature radius may lead to accidents and complications, such as perforations, transportation, steps and endodontic instrument fractures (2,3). Endodontic NiTi rotary and reciprocating files are safe instruments for canal preparation, even in severely curved root canals and decreasing the working time (4,5). In recent years, several novel thermomechanical processing and manufacturing technologies have been developed to optimize the microstructure of NiTi alloys, in order to improve their mechanical properties and root canal preparation quality (6).

The ProTaper Gold (PTG) (Dentsply Maillefer, Ballaigues, Switzerland) rotary system is a new thermally treated instrument that has the same features of the ProTaper Universal (Dentsply Maillefer, Ballaigues, Switzerland) with an advanced thermal treatment, named Gold treatment. The instruments are submitted to a complex heating-cooling proprietary treatment that results in a visible titanium oxide layer on the surface of the instrument, creating a shape memory alloy. A study has demonstrated their increased flexibility and fatigue fracture resistance and better root canal preparations (7).

Recently, a new generation of single-file Reciproc instruments were launched on the market, the Reciproc Blue (RB; VDW, Munich, Germany). This reciprocating system has the same instruments features of the conventional Reciproc M wire, but with thermomechanical blue treatment. Studies have affirmed their superiority in relation to the stability of the alloy in the martensitic phase under clinical conditions, enhanced mechanical properties, and reduction in shape-memory (6,8,9).

The aim of the study was to evaluate the shaping ability of two systems: one rotary (PTG) and the new reciprocating type (RB) in the preparation of moderately curved canals in the mesial roots of mandibular molars, by means of micro-computed tomographic (micro-CT). The null hypothesis was that there would be no difference between the two instrumentation systems in relation to changes in the 3D geometry, pointing out the dentin removed, remaining dentin thickness, increase in root canal volume, structure model index (SMI), degree of canal transportation, and centering ability.

## Material and Methods

-Selection of teeth

Ethical approval was granted by the local institutional ethics committee (protocol no. 60244116.6.0000.5137). For the study 20 human mandibular first and second molars (mesial canals) were used, with completely formed apices, of a previous sample of 108 specimens, which were stored in 0.1% Thymol solution. The inclusion criteria were: specimens with a length (21-22 mm), moderately curved (100-200) according to the Schneider method (11); curvature radius smaller than 10 mm, according to Schafer *et al.* 2002 (12), presenting independent canals and foramina. The root canal volume values were calculated for anatomic pairing of volume and SMI in order to diminish the bias of the study.

-Micro-CT Scanning

To capture the images, the SkyScan 1174 microtomograph (SkyScan, Kontich, Belgium) was used. The specimens were placed in silicone molds to allow the samples to be scanned in the same position after each step. The teeth were scanned at 47kV, 830 µA and 3600 rotation with an 0.80 rotation step, with voxel size of 16.8 µm. The images obtained were reconstructed by using the NRecon v.1.6.3 software (Bruker micro-CT).

-Root canal preparation in both groups

An experienced endodontist prepared all the specimens. Surgical access was gained with diamond burs. Afterwards, a size 10 C Pilot (VDW, Munich, Germany) was introduced for initial exploration of the canal. For lighting and magnification, an optical operating microscope (Alliance, São Paulo, Brazil) was used at 25x magnification, through which the tip of the instrument was visualized at the outlet of the major foramen, thus the working length (WL) was calculated at 1mm short of the foramen. The X Smart plus (Dentsply Maillefer, Ballaigues, Switzerland) electric motor was used for instrumentation. Each instrument was used in only one specimen. Between each change of instrument, the canal was irrigated with 5 mL of 5.25% sodium hypochlorite (NaOCl) (Lenza Farmacêutica, Belo Horizonte, Brazil) in a disposable syringe with 30-G NaviTip needles (Ultradent, South Jordan, UT, USA). Final irrigation was performed with 5 mL of 17% EDTA for 2 minutes followed by 5 mL of 5.25% NaOCl. The root canals were dried with absorbent paper points (Dentsply Maillefer, Ballaigues, Switzerland). The specimens were divided into two groups with similar characteristics as regards volumes to ensure greater homogeneity between the groups. The instrumentation techniques were performed in accordance with the manufacturers’ instructions.

PTG: The instruments were used at a speed of 250 rpm and specific torques visualized on the motor screen. Manual instruments size 10 C Pilot (VDW), 15 and 20 K-file (Dentsply Maillefer, Ballaigues, Switzerland) were used in the two thirds of the WL; afterwards the instruments S1(18/.02) and SX (19/.035) were used at the same measurement, and subsequently the manual instrument size 10 C Pilot (VDW) for performing the glide path. After this, the remaining instruments S1 (18/.02), S2 (20/.04), F1 (20/.07) and F2 (25/.08) were used up to the WL.

RB: Before used, the size 10 C Pilot (VDW) was used for initial exploration of the canal, performed with the particular Reciproc program. After this, instrument R 25/.08 was used in slow in and out movements with maximum amplitude of 3mm until the cervical and middle thirds were attained. Then the size 10 C Pilot (VDW) was again introduced to perform the glide path and then the operator advanced with instrument R 25/.08 up to the WL.

For post-operative scanning, the same parameters of pre-operative stage were used.

-Two-and Tri-dimensional Evaluation

The analyses were divided into two stages. One two-dimensional, in which the cross-sections were used, comparing the pre- and post-operative images, to evaluate transportation and centralization of the canal. The other form of analysis was tri-dimensional, to evaluate the pre- and post-instrumentation SMI; apical and total volumes of the canals, by using the CTAn software (CTAn, version1.8.1.5, Skyscan, Kontich, Belgium). This measurement was obtained between the 1st mm apical and the last mm below the furcation area. The apical volume measurement was calculated from the analysis of the measurements obtained between the 1st mm and 3rd mm of the root apex. The pre- and post-instrumentation images were superimposed for realignment, using the 3D register function of the DataViewer software v1.5.2 (Bruker-microCT, Kontich, Belgium).

-Root canal Transportation

Three (3) axial cross-sections were analyzed in the cervical (2mm below the furcation area), middle and apical (final 1mm of the root apex) thirds, with the CTAn software by means of the measurement tool. Canal transportation was calculated in millimeters using the formula (X1-X2)-(Y1-Y2) described by Gambill; Alder; Del rio, 1996 (13). According to this formula, a result of 0 indicated no canal transportation. A positive value represented mesial movement, whereas a negative value represented distal movement or movement toward the furcation (14).

-Centering Ability 

The centering ratio, which means the capacity of an instrument to remain in a central position within the root canal, was calculated in the same axial cross-sections, using the values obtained during the measurements for evaluating canal transportation. The formula used for the ratio was (X1-X2) to (Y1-Y2) (13). According to this formula, value 1 indicated the optimal centering ability (15).

-Statistical Analysis

The D’Agostino-Pearson was used to evaluate the normality. The Student’s-t test was used to evaluate differences between PTG and RB in each measurement evaluated. The level of significance was set at 5%. The analyses were performed using the GraphPad Prism Software (GraphPad Software, San Diego, California, USA).

## Results

**Figure 1:**
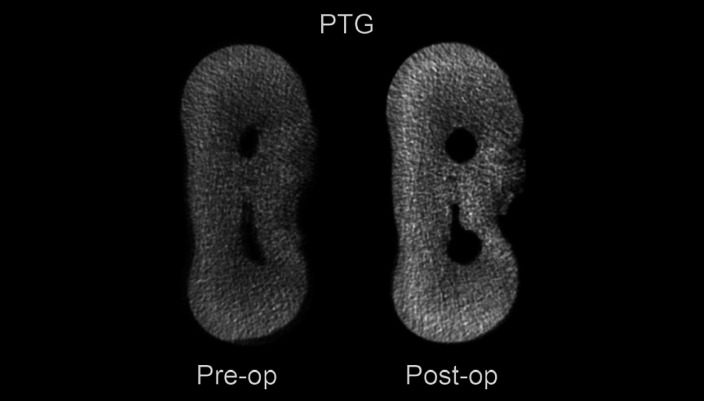
Micro-Ct images of the amount of dentin remaining on the Protaper Gold instrument.

**Figure 2:**
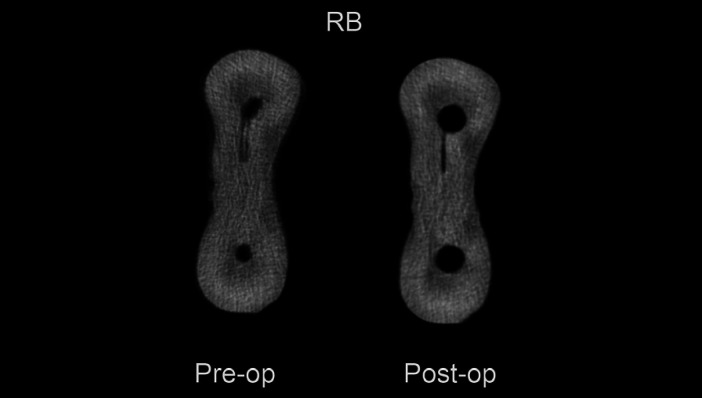
Micro-Ct images of the amount of dentin remaining on the Reciproc Blue instrument.

The Structure Model Index (SMI), removed dentin and canal volume increase were evaluated and no significant diferences were observed between RB and PTG (*P*>0.05), except for the mesiobucal canal in SMI. (Table 1). Both systems presented deviations in the cervical third (Figs. 1,2). There were no significant differences between RB and PTG in root canal transportation in the cervical, middle and apical regions (*P*>0.05) (Table 2). As regards centering ability, there were no significant differences between RB and PTG in the cervical and middle regions of MB and ML canals; and in the apical region of the ML canal (*P*>0.05). However, there were significant differences in the apical region of the MB canal, where the RB instruments were superior when compared with the PTG (*P*<0.05).

**Table 1:**
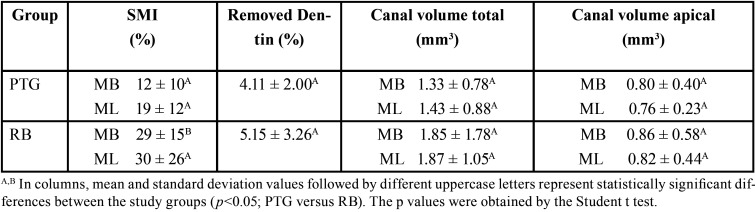
Structure Model Index (SMI), Removed Dentin and Canal Volume Increase (mean ± standard deviation).

**Table 2:**
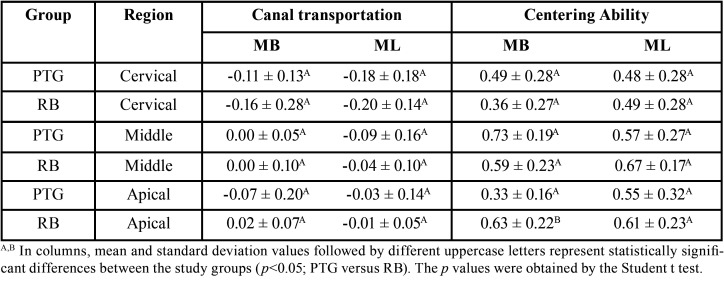
Root canal Transportation and Centering Ability Values Observed (mean ± standard deviation).

There were no significant differences in dentin removal between RB and PTG in the cervical, middle and apical regions (*P*>0.05). RB obtained the highest mean of dentin removal from the distal surface of the ML canal in the cervical region (Table 3).

**Table 3:**
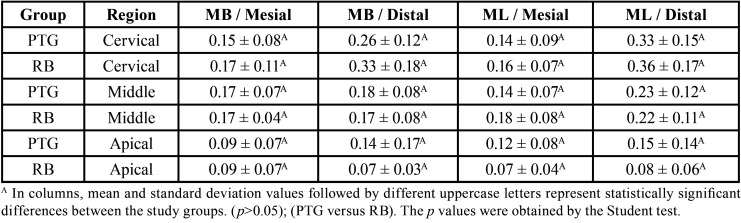
Dentin removal (mm) after preparation of the root canals. (mean ± standard deviation).

Concerning the remaining dentin thickness. There was no significant difference between RB and PTG (*P*>0.05), except on the mesial surface in the cervical region of MB canal; on the mesial surface in the cervical and middle regions of ML canal, and on the distal surface of the middle region of ML canal, where RB was observed to have a superior cutting capacity (*P*<0.05) (Table 4). The lowest remaining dentin thickness mean was obtained by PTG on the distal surface of ML canal in the apical region, (Table 3).

**Table 4:**
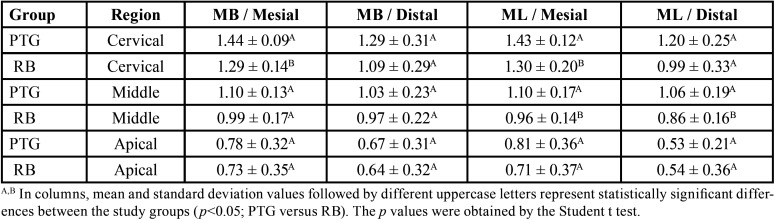
Remaining dentin thickness (mm) after preparation of the root canals. (mean ± standard deviation).

## Discussion

One of the aims of root canal preparation is to maintain its original anatomy in a conservative and safe manner (16) however, errors may occur, such as transportations, deviations and steps, which may compromise the success rates of endodontic therapy (17). This study compared the capacity of PTG and the RB system in maintaining the original trajectory in moderately curved mesial canals of mandibular molars. The null hypothesis that there would be no difference in the performance of the two systems was confirmed, except with regard to the centering ability of the apical third, SMI in canal MB.

**Figure 3:**
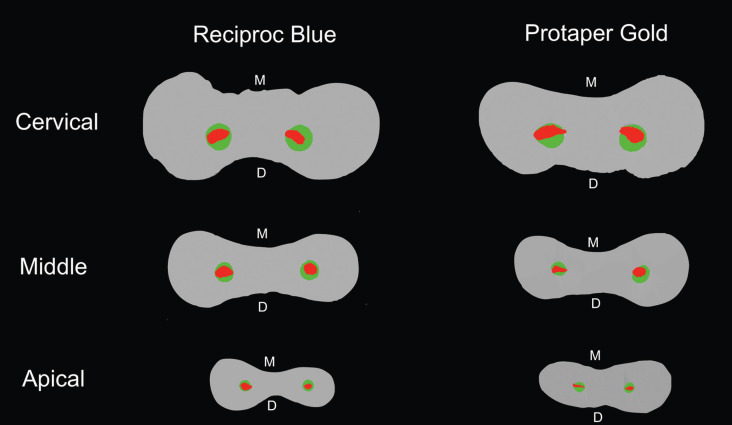
Representative cross sections of superimposed root canals before (red) and after (green) preparation in cervical, middle and apical thirds. The image shows greatest dentin removal toward the furcation or distal (D) area, compared with mesial (M) area, especially in the cervical third.

As regards the amount of remaining dentin in the furcation region, both systems presented deviations in the cervical and middle thirds (Fig. 3). Nevertheless, no specimen presented perforation. The PTG system showed greater wear in the furcation region, probably because of the increase in the tapering of the instruments and less flexibility than those of RB. Another important factor that reinforces this is that there is a greater volume of dentin on the mesial wall, which tends to force the instrument in the distal direction in this region (18).

Previous studies have shown that thermally treated instruments produce more centralized root canal preparations, with lower capacity for transportation (6). Apical transportation of over 0.3 mm may negatively influence the sealing capacity of the filling material (19). In spite of there being no significant differences, three canals in which the PTG instruments were used presented apical transportation values of over 0.3 mm and this was not observed in any canal in Group RB. These results could be explained due to the different cross-sectional design and the thermal treatments of the PTG and RB. However, Souza-Neto *et al.*, (2018) analyzes, where they already assess that apical transportation exceeds the limit of 0,3mm, especially in reciprocating instrumentation (26). In other study comparing Wave One 25.08 and Reciproc 25.08, obtained no differences in terms of area, perimeter, volume, surface area or transportation (27). The cross-sectional design, diameter of the core and thermal treatment affect the flexibility of the NiTi instruments, which can affect the transport of the root canal during root canal shaping (6,20).

Endodontic preparations should be more conservative as possible in order to not weaken the root walls. Clinically, a 0,2 milimeters removal seems to be adequate for root canal preparation of molars (21). In our study, both PTG and RB and remaining dentin thickness in some regions systems yielded root canal preparations within this limit in the majority of the cases. However, the wear was always directed towards the distal region. All the measurements in both groups showed a thickness of the remaining dentine above 0,5 milimeters after root canal preparation, thus being deemed satisfactory and safe in the three thirds assessed.

Furthermore, there are differences between the instruments in relation to kinematics, in which PTG is used in continuous rotation and RB with reciprocal motion. Study have pointed out that the reciprocating movement presents advantages, because in addition to running a smaller angular distance, and consequently being subject to reduced stresses, it generates an increase in cyclic fatigue resistance (22). The application of reciprocating motion during instrumentation did not result in increased apical transportation when compared with continuous rotation motion (20,21).

In this study, the 3D geometry and shape of the cross-section of the mesial canals were evaluated using the SMI which is a morphometrical parameter and involves a measurement of surface convexity in a tridimensional structure. It evaluates the capacity of a preparation technique to produce more rounded canals (23). The RB instruments had a significantly higher SMI value than the PTG instruments in canal MB; so, they obtained more rounded preparations, which could positively influence the filling of root canals that have a greater complexity. The authors could attribute the cross-section of the RB instrument in particular to these more circular preparations, or to the canals; although they had similar volumes, they could have been more flattened and therefore have influenced the results.

In this study, the RB instruments were more effective in the centralization of the MB canals in the apical third, demonstrating greater safety and effectiveness in relation to the root canal preparation (2,3). The blue thermal treatment provided an increased flexibility of the instruments (9,24), and thus the hypothesis of more centralized preparations and reduction of the shape memory effect (8,10). However, there is a systematic review study, which concluded that there is no significant difference in relation to the rotary and reciprocation motions, in the transportation and centering ability of root canals (25).

Within the limits of this study, it was concluded that both systems were efficient and safe for performing preparation of the moderately curved root canals of mandibular molars. However, the RB instruments produced more circular and better centralized canals in the apical third of the MB canal, and showed a superior cutting action when compared with the PTG instruments. Further studies will be necessary, mainly with different types of endodontic instruments, with new alloys and thermomechanical treatments.
